# Generation of orthotopic patient-derived xenografts from gastrointestinal stromal tumor

**DOI:** 10.1186/1479-5876-12-41

**Published:** 2014-02-10

**Authors:** Jason K Sicklick, Stephanie Y Leonard, Michele L Babicky, Chih-Min Tang, Evangeline S Mose, Randall P French, Dawn V Jaquish, Carl K Hoh, Michael Peterson, Richard Schwab, Andrew M Lowy

**Affiliations:** 1 Division of Surgical Oncology and Department of Surgery, Moores UCSD Cancer Center, University of California, San Diego, 3855 Health Sciences Drive, Mail Code 0987, La Jolla, CA 92093-0987, USA; 2 Division of Nuclear Medicine, Moores Cancer Center, and the UCSD In Vivo Cancer & Molecular Imaging Program, University of California, San Diego, La Jolla, CA USA; 3 Department of Pathology, University of California, San Diego, La Jolla, CA USA; 4 Division of Medical Oncology and Department of Internal Medicine, Moores UCSD Cancer Center, University of California, San Diego, La Jolla, CA USA

**Keywords:** GIST, Imatinib, KIT, NOD-scid, NOD-scid gamma, PDX, Sarcoma, Targeted therapy

## Abstract

**Background:**

Gastrointestinal stromal tumor (GIST) is the most common sarcoma and its treatment with imatinib has served as the paradigm for developing targeted anti-cancer therapies. Despite this success, imatinib-resistance has emerged as a major problem and therefore, the clinical efficacy of other drugs has been investigated. Unfortunately, most clinical trials have failed to identify efficacious drugs despite promising *in vitro* data and pathological responses in subcutaneous xenografts. We hypothesized that it was feasible to develop orthotopic patient-derived xenografts (PDXs) from resected GIST that could recapitulate the genetic heterogeneity and biology of the human disease.

**Methods:**

Fresh tumor tissue from three patients with pathologically confirmed GISTs was obtained immediately following tumor resection. Tumor fragments (4.2-mm^3^) were surgically xenografted into the liver, gastric wall, renal capsule, and pancreas of immunodeficient mice. Tumor growth was serially assessed with ultrasonography (US) every 3-4 weeks. Tumors were also evaluated with positron emission tomography (PET). Animals were sacrificed when they became moribund or their tumors reached a threshold size of 2500-mm^3^. Tumors were subsequently passaged, as well as immunohistochemically and histologically analyzed.

**Results:**

Herein, we describe the first model for generating orthotopic GIST PDXs. We have successfully xenografted three unique KIT-mutated tumors into a total of 25 mice with an overall success rate of 84% (21/25). We serially followed tumor growth with US to describe the natural history of PDX growth. Successful PDXs resulted in 12 primary xenografts in NOD-scid gamma or NOD-scid mice while subsequent successful passages resulted in 9 tumors. At a median of 7.9 weeks (range 2.9-33.1 weeks), tumor size averaged 473±695-mm^3^ (median 199-mm^3^, range 12.6-2682.5-mm^3^) by US. Furthermore, tumor size on US within 14 days of death correlated with gross tumor size on necropsy. We also demonstrated that these tumors are FDG-avid on PET imaging, while immunohistochemically and histologically the PDXs resembled the primary tumors.

**Conclusions:**

We report the first orthotopic model of human GIST using patient-derived tumor tissue. This novel, reproducible *in vivo* model of human GIST may enhance the study of GIST biology, biomarkers, personalized cancer treatments, and provide a preclinical platform to evaluate new therapeutic agents for GIST.

## Background

Gastrointestinal stromal tumor (GIST), the most common gastrointestinal mesenchymal tumor, afflicts 12-20 patients per million annually [1]. Unlike many other cancers, the genomic and molecular events driving GIST are well characterized. These include mutations in several protein kinase genes including KIT, PDGFR*α*, and BRAF that are known to regulate fundamental processes in oncogenesis including tumor proliferation, metastasis, neo-vascularization, and chemo-resistance [2-4]. GIST has served as a paradigm for the development of targeted cancer therapies because inhibition of KIT and PDGFR*α* has resulted in therapeutic benefit. At present, the first-line treatment for patients with metastatic, unresectable or resected high-risk GIST is imatinib (Gleevec, Novartis, Basel, Switzerland) [5-7], a small molecule inhibitor of tyrosine kinases including KIT (c-KIT, CD117) and BCR-ABL. This drug has been shown to have profound therapeutic benefit with a favorable toxicity profile. Because of these qualities, imatinib is often cited as the prototype for targeted therapeutics development.

Beyond our knowledge that KIT mutations drive GIST sarcomagenesis, it is now known that specific KIT mutations are both prognostic and predictive of responses to the current kinase inhibitors. For example, KIT Exon 9 mutations are associated with more aggressive phenotypes and imatinib insensitivity as compared to KIT exon 11 mutations [4,8]. Secondary resistance to imatinib, which occurs in half of patients after 20 months of therapy, is most commonly caused by acquired, non-randomly distributed single nucleotide KIT mutations within the ATP binding pocket (exons 13 and 14) and the kinase activation loop (exons 17 and 18) [9-12]. Sunitinib (Sutent, Pfizer, New York, NY), a multikinase inhibitor with activity against PDGFR, VEGFR and KIT, is employed as second line therapy for GIST. Clinical trials have shown that in imatinib-resistant cases, only 12-19% of sunitinib-treated patients have significant responses [13,14]. These anti-GIST therapies were developed based upon efficacy data *in vitro* or *in vivo* using subcutaneous models of tumor implantation. However, once a patient progresses on sunitinib, treatment options are limited as evidenced by two recent, large clinical trials which reported on the efficacy of dasatinib (Bristol-Myers Squibb, New York, NY), a combined Src and BCR-ABL inhibitor, and regorafenib (Bayer, Pittsburgh, PA), a combined VEGFR2 and TIE2 inhibitor [15,16]. Dasitinib failed to show any benefit in this patient population while in the Phase III GRID trial of regorafenib, 62% of patients developed resistance to the drug, and consequently disease progression by the sixth month of therapy [17]. This highlights the urgency for developing more effective agents to treat GIST [18], as well as more broadly applicable preclinical models to accomplish this goal.

Despite the importance of preclinical studies on GIST tumorigenesis and resistance mechanisms, there are currently limited model systems for studying this disease *in vitro* and *in vivo*. For instance, two GIST cell lines with KIT exons 11 and 13 mutations have been reported in the literature; [19,20] however, the second most common KIT mutation (e.g., exon 9) lacks a corresponding cell line for *in vitro* assays. Moreover, there are no cell lines which contain any exon 14 or 18 mutations while most of the common exon 17 mutations are not present in cell lines except with overexpression vectors often used in non-GIST lines, such as BaF3 cells. In addition, no cell lines exist which contain either PDGFR*α* mutation/deletions/insertions or BRAF^V600E^ mutations that also cause GIST. Regarding mouse models of GIST, subcutaneous (SQ) xenografts have been utilized as the prototype in nude mice [21-23]. However, because tumor growth or responses to drug treatment observed in SQ xenograft models are often different from those observed in an orthotopic environment, two groups have developed transgenic mouse models of GIST. Rubin and colleagues identified a KIT^K641E^ mutation (exon 13) in sporadic human GISTs and in the germ line of familial GIST syndrome patients [24]. They then generated homozygous and heterozygous *KIT*^K641E^ transgenic mice that develop cecal GISTs with complete penetrance. However, in humans, cecal (e.g., colonic) GISTs are quite rare, suggesting that this model does not completely recapitulate the human disease. Additionally, Besmer and colleagues developed a second model via a knock-in strategy by introducing a KIT exon 11 mutation (V558^Δ/+^) into the mouse genome [25]. While the latter transgenic model is more representative of the human disease, it only embodies a mutation that is well studied, evaluable in the GIST-T1 cell line, and sensitive to imatinib.

Despite the aforementioned models, there remains a gap in our ability to predict effective drugs or study the biology of the less frequent, but often drug-resistant, gene mutations in GIST. Therefore, our goal was to develop a reproducible, orthotopic patient-derived xenograft model of GIST. This novel model for studying GIST *in vivo* recapitulates the intra-abdominal microenvironment in which GIST arises and allows for the study of the increasingly appreciated heterogeneity in the biology of GIST. It is our hope that this model may serve as a valuable resource for personalized cancer therapy and the evaluation of new therapeutic agents for GIST.

## Materials and methods

### Animal studies

NOD-scid (NS) and NOD-scid IL2Rgamma^null^ (NOD-scid gamma, NSG) mice at 8-10 weeks of age were obtained from the Jackson Laboratory (Bar Harbor, Maine). NS homozygous mice harboring a spontaneous Prkdc^scid^ mutation (commonly referred to as scid) are a model for severe combined immune deficiency characterized by an absence of functional T cells and B cells, hypogammaglobulinemia, lymphopenia, and a normal hematopoietic microenvironment. NSG mice combine the features of the NOD/ShiLtJ background, the severe combined immune deficiency mutation and an IL2 receptor gamma chain deficiency. As a result, this NSG strain (NOD.Cg-Prkdc^scid^ Il2rg^tm1Wjl^/SzJ), lacks functional T cells, B cells, and NK cells, as well as is deficient in cytokine signaling. Consequently, this NSG strain performs better in engraftment of human hematopoietic stem cells and peripheral-blood mononuclear cells than any other published mouse strains [26-31]. Moreover, these recent publications have demonstrated this strain’s utility in the study of solid tumor xenografts and cancer stem cell engraftment.

All research mice were maintained in a barrier facility under HEPA-filtered air with food and water available *ad libitum*. Food, water and cage bedding were sterilized prior to use. Temperature (20-21°C), humidity (50-60%) and 12-hour light-dark cycle were controlled. Animals were manipulated under sterile conditions during surgery. Animal experiments fulfilled National Institutes of Health and University of California, San Diego (UCSD) requirements for humane animal care. The UCSD Institutional Animal Care and Use Committee approved experimental methods.

### Sourcing of human tumor tissue

Tumor acquisition/banking is routinely performed for all GIST operations under our Institutional Review Board (IRB) approved protocol (#090401). Written informed consent was obtained from all patients prior to sample collection. Three patients with KIT-mutated GIST underwent operations in 2011. All patients’ demographics were listed in Table 1. The tumor tissue for xenografts was obtained at the time of tumor resection after a pathologist acquired tissue that was needed for the patient’s routine clinical care and confirmed the histologic diagnosis. Additional tissue was banked in our biorepository. Excess fresh tumor was used for immediate xenografting into mice. All surgically resected tumor fragments were stored in sterile specimen cups and expeditiously transported from the operating room to our laboratory on ice. Staining for clinical diagnosis included hematoxylin and eosin (H&E), KIT and DOG-1. Genetic materials derived from tumors were analyzed by ARUP Laboratories (Salt Lake City, UT) for KIT and PDGFRα mutations.

**Table 1:** Patient demographics

	**Number**
**Age**	
Mean	62 years
Range	46 – 72 years
**Gender**	
Male	2
Female	1
**Location of primary tumor**	
Stomach	0
Small bowel	3
Duodenum	1
Jejunum	1
Ileum	1
**Presentation**	
Abdominal pain	2
Bleeding	1
Recurrence/metastasis on CT scan	1
**Previous imatinib therapy**	
Yes	1
No	2
**Primary tumor size**	
Mean	19.2 cm
Range	6.5 – 38.0 cm
**Mitotic index (mitoses per 5 mm**^**2**^**)**	
Mean	32.7
Range	10 – 65
**Peritoneal metastases**	
Yes	2
No	1
**KIT exon mutational analysis**	
Exon 9	2
Exon 11	1
Exon 13/14	0
Exon 17/18	0

### Implantation of patient-derived xenografts

Tumor was dissected into 2×2 mm fragments (4.2 mm^3^) and placed in a petri dish kept on ice containing sterile, antibiotic-free DMEM media (Mediatech, Manassas, VA) until implantation. NS and NSG mice were anesthetized with intraperitoneal injection of ketamine:xylazine cocktail (60-mg/kg : 10-mg/kg). They were then placed in the supine position on a warm pad to maintain body temperature. Once mice were sedated, the abdominal wall was shaved and cleansed with 70% alcohol and betadine. A 1-2-cm midline incision was made through the skin, fascia and peritoneum. Surgical sutures (6-0 silk) were used to implant 2×2 mm tumor fragments onto the livers, gastric walls, renal capsules, or lesser sacs (peri-pancreatic area). Organs implanted with tumor fragments were returned to the abdomen and the peritoneum and the skin were closed with 6-0 Prolene suture. A total of 14 animals underwent initial tumor implantation of freshly dissected human tumor tissues.

Mice were monitored daily for 5 consecutive days after surgery with particular attention paid to animal distress, wound dehiscence, and signs of infection. Thereafter, they were examined 2-3 times per week. Three researchers (SL, CT and EM) assessed tumor progression by palpation twice a week. Tumor progression was also evaluated by ultrasound every 3-4 weeks as described in the “Tumor Imaging” section. Animals were euthanized based on either tumor volume (threshold 2500 mm^3^) as determined by ultrasound or clinical status during the observation period as specified in our IACUC-approved protocol. A necropsy was performed on the animals after euthanasia to assess the presence and distribution of tumors. Tumors were harvested and fixed in 10% formalin for histological and immunohistological analyses. Harvested tumors were also subject to serial passages into additional 11 mice. All 3 patient-derived xenografts were successfully passaged up to twice in order to determine the ability to perpetuate and expand these tumors for extended periods of time. This provides the potential for developing a model that is based upon a small amount of available tumor, which can be utilized for current and future studies. Mouse characteristics for tumor implantation are listed in Table 2.

**Table 2:** Mouse characteristics for generation of GIST patient-derived xenografts

	**Number**	**Percent (%)**
**Age at implantation**		
Mean ± standard deviation	9.1 ± 7.3 weeks	
Range	5.7 – 30.6 weeks	
**Gender**		
Male	8	32.0%
Female	17	68.0%
**Genotype**		
NOD-scid (NS)	10	40.0%
NOD-scid gamma (NSG)	15	60.0%
**Tumor development**		
Yes	21	84.0%
No	4	16.0%
**Passages**		
0 (Primary, successes/total)	11/14	85.7%
1 (Successes/total)	5/6	83.3%
2 (Successes/Total)	5/5	100.0%
**Location of implantation**		
Successful implantations/total	21/25	84.0%
Liver (successes/total)	9/10	90.0%
Renal capsule (successes/total)	9/10	90.0%
Lesser sac (successes/total)	2/3	66.7%
Gastric wall (successes/total)	1/2	50.0%

### Tumor imaging

Tumors were serially imaged with Visual Sonics Vevo 770 ultrasound machine (VisualSonics Inc, Toronto, Canada) every 3-4 weeks by a single, experienced ultrasonographer. Mice were kept anesthetized using continuous isoflurane inhalation. Prior to ultrasonography, abdominal wall hair was removed from the skin overlying the tumor implant area with clippers and hair removal cream. Their skin was then covered with an aqueous ultrasonic gel and a high frequency transducer (RMV706) at 20-60 MHz range was used for imaging. Tumor detection was recorded as cine loops of ultrasound images. Digital images were reviewed to select the tumors’ largest cross-sectional diameters (e.g., length and width) from a single image frame. Tumor volume was calculated as (**π**/6) × (length × width)^3/2^[32].

Two animals were also evaluated with Positron Emission Tomography (PET, GE eXplore Vista DR PET scanner). Mice were anesthetized by isoflurane inhalation and placed on an imaging bed controlled by a computer in order to insert into the ring-type gantry. They were then administered a radiopharmaceutical fluorodeoxyglucose-[18F] (F-18 FDG) delivered by tail vein injection. F-18 FDG is used for the assessment of glucose metabolism, and therefore serves as an indicator for high metabolic activity of tissue, such as malignant tumors. One mouse was given 250 μCi in 113 μL while another mouse received 395 μCi in 200 μL. A sequence of successive whole-body images was acquired in the 3D mode using the system software. DICOM images were analyzed by an experienced nuclear medicine radiologist (CKH) for tumor standardized uptake values (SUVs).

### Histopathology and immunohistochemistry

Serial sections of formalin-fixed, paraffin-embedded (FFPE) resected xenografts, 10-μm thick, were used for histopathology analyses by H&E staining. For all tumors, the histological diagnoses were confirmed under light microscopy by an experienced pathologist. Sections for immunohistochemical staining were treated twice with 0.3% Triton X-100 (Sigma-Aldrich) in PBS for 10 minutes and then in 0.3% hydrogen peroxide solution in order to block endogenous peroxidase activity. The sections were then blocked with serum followed by an Avidin-Biotin blocking reagent (Vector Laboratories; Burlingame, CA) in order to inhibit non-specific binding in the tissue. The sections were then incubated with polyclonal rabbit anti-human CD117 (c-KIT) antibody (1:50, Dako North America, Carpinteria, CA) overnight at 4°C. Sections were next incubated with biotinylated secondary antibody and ABC reagents of the Vectastain Elite Universal ABC kit according to the manufacturer’s instructions (Vector Laboratories). The secondary antibody was detected using the Avidin-Biotin-Peroxidase method with 3,3′-diaminobenzidine as the substrate (Vector Laboratories). Negative controls were performed by omitting the primary antibody and/or using isotype control antibody.

### Statistical analysis

Comparisons between groups were performed using Prism 4 (GraphPad, La Jolla, CA). Results are reported as mean ± standard deviation (s.d.) or standard error of the mean (s.e.m.) as appropriate. Comparison data were analyzed for significance using the Student’s t-test, ANOVA, and Bonferroni’s multiple comparison test. Statistical significance was accepted at the 5% level and statistical trends were accepted at the 10% level.

## Results

### Sourcing human GISTs

**Figure 1:**
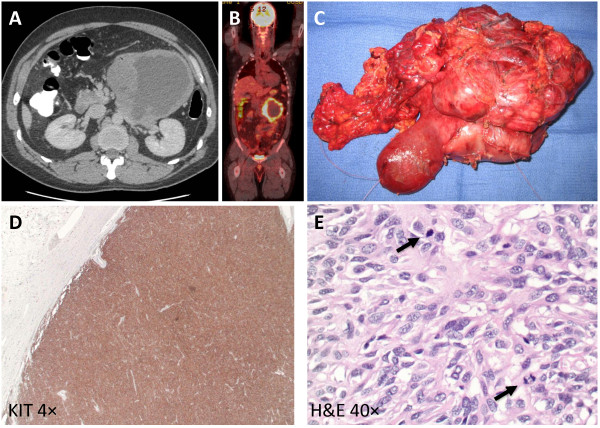
**Resection of a 13-cm GIST from a 46-year old man that presented with abdominal pain. A**. CT of the abdomen demonstrates a heterogeneous mass in the left abdomen. **B**. PET-CT demonstrating an FDG-avid mass in the left upper quadrant. **C**. Gross specimen following resection of the third and fourth portions of the duodenum and proximal jejunum where the tumor was arising. **D**. Pathology consistent with a GIST with strong KIT immunostaining. **E**. H&E staining demonstrating 23 mitoses (arrows) per 50 high power fields.

To our knowledge, only subcutaneous (SQ) GIST xenografts have been performed in mice. We hypothesized that human GISTs could be intraperitoneally (IP) xenografted into immunodeficient mice in order to better recapitulate the biology of GIST, a disease which tends to metastasize to the liver and peritoneum, but not the soft tissue of the flank. KIT-mutated GIST tissue from three patients was used for xenografts in this study (Table 1). This included tumors from 2 male patients and 1 female patient with mean age of 62. Their primary tumors were all found in the small bowel. One patient had a clinical presentation of worsening abdominal pain while the second patient presented with acute onset abdominal pain due to intratumoral bleeding. The third patient had GIST recurrence and metastatic tumors detected by CT scan. Only the latter patient had previously received imatinib therapy. The mean tumor size was 19.2 cm (range: 6.5 - 38.0 cm) with an average mitotic index (mitoses per 50 high-power fields equals mitoses per 5 mm^2^) of 32.7 (range: 10 - 65). Based upon pathological examination, one patient had stage IIIB and the other two patients had stage IV GIST with peritoneal involvement. Genetic sequencing analyses revealed that two tumors had *KIT* exon 9 mutations and one tumor had an exon 11 mutation. Herein, we present a representative case of a 46-year old male patient (Figure 1). The patient was first examined by CT scan and found to have a heterogeneous tumor mass in the left upper quadrant of the abdomen (Figure 1A) which was FDG-avid on PET-CT scan (Figure 1B). He underwent surgical resection of a 13.0 × 11.0 × 10.0 cm GIST removed from the fourth portion of the duodenum and the proximal jejunum (Figure 1C). Histologically the tumor tissue had strong KIT (Figure 1D) and DOG-1 staining (data not shown), consistent with GIST. This tumor had mixed spindle cell and epithelioid histology, as well as a mitotic index of 23 per 50 high-power fields (hpf) (Figure 1E). Similarly, the other two tumors also had high-risk features.

### Development of GIST PDXs

To develop a novel xenograft model of GIST *in vivo*, fresh human tumor tissues were implanted within immunodeficient mice. We employed a midline laparotomy to suture 2×2 mm tumor fragments into the abdominal viscera of NS (N = 10) and NSG (N = 15) mice. This included 14 primary xenografts and 11 passaged xenografts. Fresh tumor tissues implanted into 14 mice were defined as Passage zero (P0). Tumor tissues were harvested from P0 mice and implanted into 6 mice as Passage 1 (P1); and subsequently another xenograft with P1 tumors was carried out in 5 mice as Passage 2 (P2). Xenografts were performed in 25 mice with an 84% (21/25) success rate which included a 4% (1/25) perioperative mortality in a P2/NS mouse. Different implantation sites were compared for xenograft efficiency. We observed tumor growth and progression in the liver (9/10), renal capsule (9/10), lesser sac (2/3), and gastric wall (1/2). There was no tumor growth in three mice with the following characteristics: P0/NSG/Kidney; P1/NSG/Liver; and P0/NS/Stomach. Detailed characteristics of the mice used for the PDXs are shown in Table 2.

### Natural history of GIST orthotopic PDXs

**Figure 2:**
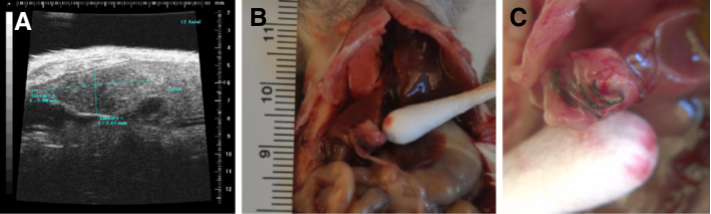
**Generation of GIST xenografts.** Tumor was xenografted into the livers, kidneys, lesser sacs and gastric walls of NOD-scid and NSG mice. Using ultrasound, we followed tumor progression. **A**. At 4 weeks, one tumor in the liver increased from 4 × 2 mm to 7 × 2.4 mm based upon ultrasound examination. **B-C**. At 7 weeks, the same mouse became distressed and we performed a necropsy with passage of the tumor into two NOD-scid mice. The tumor is noted at the end of the cotton swab. 6-0 silk suture material can be seen at the site of implantation.

**Figure 3:**
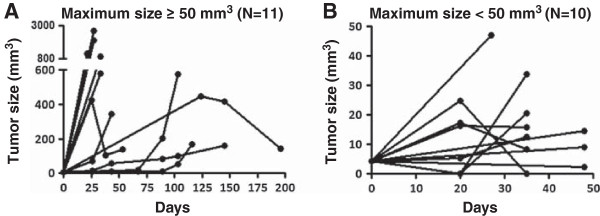
**Natural history of xenograft tumor progression based upon serial ultrasound imaging.** Based upon maximum tumor size achieved, tumors were sorted into two groups with distinct tumor growth patterns. **A**. Tumors with a maximum tumor size ≥ 50 mm^3^ (N = 11). **B**. Tumors with a maximum tumor size < 50 mm^3^ (N = 10).

Given the intra-abdominal location of tumors, standard calipers cannot be employed to monitor tumor growth. Therefore, in order to monitor the natural history of tumor progression, ultrasound (US) imaging was conducted every 3-4 weeks after implantation. As shown in Figure 2A, one tumor in the liver reached 7 × 2.4 mm in size as determined by US at 4 weeks. By 7 weeks, the same mouse had to be terminated due to poor health. The tumor was harvested (Figure 2B-C) and passaged into additional NS mice. In the entire cohort, PDX tumor size (as determined by US imaging) at 2.9-33.1 weeks (median 7.9 weeks) averaged 473 ± 695 mm^3^ (median 199 mm^3^, range 12.6-2682.5 mm^3^). Every surviving mouse with a PDX received at least 2 serial US studies (mean 2.5 ± 1.7, median 2, range 0 - 7) in order to monitor the natural history of their tumor growth. Based upon maximum tumor size achieved, we could sort tumors into two groups with distinct tumor growth patterns (Figure 3). In general, tumors with a maximum tumor size ≥ 50 mm^3^ (Figure 3A, N = 11) tended to be faster growing than those with a maximum tumor size < 50 mm^3^ (Figure 3B, N = 10). However, in the former group, there were outliers that began growing slowly but later achieved a larger final tumor volume.

**Figure 4:**
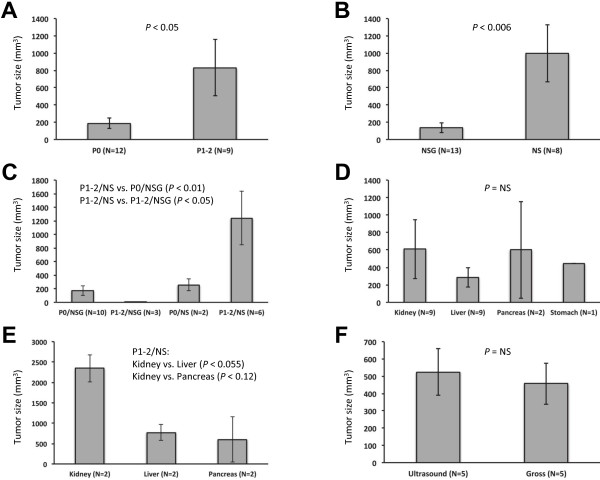
**Maximum xenograft size depends upon tumor passage, immunodeficient mouse type, and tumor implantation site. A**. Comparison of tumor passages (P0 vs. P1-2). **B**. Comparison of NSG and NS immunodeficient mice. **C**. Subgroup comparison of passages plus NSG or NS immunodeficient mice. **D**. Comparison of tumor implantation sites. **E**. Comparison of tumor implantation sites in P1-2/NS mice. **F**. Comparison of gross tumor size at necropsy with ultrasound findings obtained within 2 weeks of death.

To further assess the how tumor passage, immunodeficient mouse type, and tumor implantation location affected maximum tumor size, we performed subgroup analyses of the 21 mice that developed tumors (Figure 4). P1-2 tumors (829.5 ± 326.1 mm^3^, N = 9) were larger than P0 tumors (187.1 ± 59.4 mm^3^, N = 12, P < 0.05, Figure 4A). NS tumors (994.1 ± 329.7 mm^3^, N = 8) were larger than NSG tumors (135.2 ± 56.7 mm^3^, N = 13, P < 0.006, Figure 4B). Furthermore, P1-2/NS tumors (1240.0 ± 392.5 mm^3^, N = 6) were larger than P0/NSG tumors (173.2 ± 69.9 mm^3^, N = 10, P < 0.01) and P1-2/NSG tumors (8.6 ± 3.6 mm^3^, N = 3, P < 0.05, Figure 4C). There was no difference between P1-2/NS tumors and P0/NS tumors (256.4 ± 87.3 mm^3^, N = 2) due to the small sample size of the latter group. Comparison of the maximum tumors sizes of the four implantation locations (Kidney: 610.9 ± 338.5 mm^3^, N = 9; Liver 284.7 ± 109.1 mm^3^, N = 9; Pancreas 601.6 ± 554.5 mm^3^, N = 2; Stomach 446.4 mm^3^, N = 1) showed no statistically significant differences due to variability within the groups, confounding factors such as passage/mouse type, or small sample sizes (Figure 4D). However, in subgroup analyses, the P1-2/NS mice kidney tumors (2347.6 ± 334.9 mm^3^, N = 2) tended to be larger than liver tumors (770.6 ± 191.5 mm^3^, N = 2, P < 0.055) and pancreas tumors 601.6 ± 554.5 mm^3^, N = 2, P < 0.12, Figure 4E). To determine the accuracy of our ultrasound findings, we compared the tumor sizes of 5 mice that died or were sacrificed within 2 weeks of their last US. There was no statistically significant difference between the groups (US: 524.3 ± 134.6 mm^3^ vs. 457.2 ± 119.4 mm^3^, P = NS, Figure 4F).

### Histological analyses of GIST PDXs

**Figure 5:**
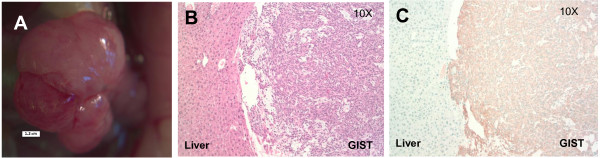
**Xenograft tumor progression is marked by tumor growth and KIT immunostaining of a spindle cell neoplasm. A**. At 21.1 weeks, this P0 mouse was sacrificed for tumor passage. The primary tumor in the liver measured 8.5 × 7 × 6.5 mm on gross examination. **B**. Blinded review was performed by a pathologist. H&E staining confirmed the presence of a spindle cell neoplasm consistent with a GIST. This tumor appears to invade into the liver parenchyma. **C**. Positive KIT immunostaining was present in the xenografted tumor but not in the adjacent non-neoplastic liver.

To investigate if PDXs maintain human GIST tumor properties after implanting tumor into mice (i.e., P0) or after passage once into additional mice (i.e., P1), six mice were sacrificed and their tumor tissues were subject to GIST histopathological analyses and KIT immunohistochemical staining. Five of the six (83.3%) maintained strong KIT staining of the tumors. It is notable that the hallmarks of tumor necrosis were not seen in the one spindle cell neoplasm lacking KIT expression. Thus, the mechanism for KIT downregulation remains unknown. Despite the presence of tumors, 4 mice were not evaluable histologically due to tissue necrosis overnight. Another 8 mice had tumors, which did not reach our set threshold size of 2500 mm^3^ for sacrifice and passage, became quite ill due to the Staphylococcal epidemic in our vivarium. We prematurely sacrificed these mice and the tumors tissues were used for passaging to additional healthy mice, leaving no tissue for additional histological analyses. However, this suggests that even in the event of an infection or illness, tumors can be salvaged for additional passaging and study. An example of a P0 mouse with GIST histopathology and KIT staining is shown in Figure 5. At 21.1 weeks, this P0 mouse had an 8.5 × 7 × 6.5 mm tumor in the liver on gross examination (Figure 5A). To verify the primary tumor histologically, serial sections of tumor tissues were stained by H&E and blindly reviewed by a pathologist (MP). It was evident that a spindle cell neoplasm was present in the primary tumor but not in the neighboring liver tissue (Figure 5B). Furthermore, in contrast to the adjacent non-neoplastic liver that lacked KIT staining, the implanted tumor had strong KIT immunostaining signals (Figure 5C).

### PET imaging of GIST PDXs

**Figure 6:**
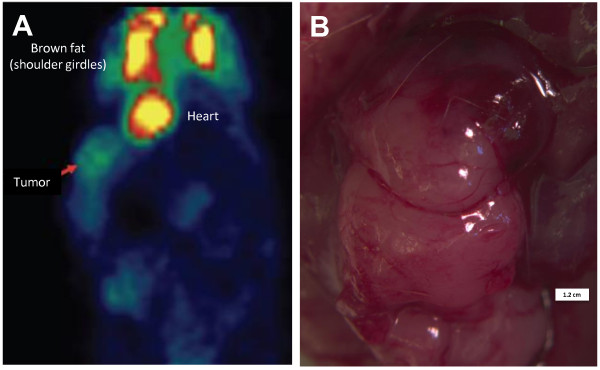
**Human GIST xenografts are FDG-avid on PET scan. A**. This P0 mouse underwent tumor xenografting into the right renal capsule. At 16.1 weeks after implantation, we performed imaging via PET scan. The arrow denotes tumor uptake of FDG (SUV_max_ 2.2, SUV_min_ 1.8, SUV_mean_ 2.0) by PET scan as indicated by the arrow in **A**. The FDG uptake in the heart and the brown fat of the shoulder girdles serve as a positive control. **B**. Gross photograph of the corresponding FDG-avid tumor measuring 12 × 10.5 mm in the same mouse.

PET scan was employed to assess xenografts for human GIST tumor properties. Two mice with tumors from the patient 1′s FDG-avid tumor (Figure 1) were evaluated with PET scan and both tumors were FDG-avid on PET. As shown in Figure 6, a P0 mouse had tumor implanted onto the right renal capsule and was subject to PET scan at 16.1 weeks. The xenograft measured 12 × 10.5 mm on gross examination (Figure 6B) and was FDG-avid (SUV_max_ 2.2, SUV_min_ 1.8, SUV_mean_ 2.0) by PET scan as indicated by the arrow in Figure 6A. The FDG uptake in the heart and the brown fat of the shoulder girdles serve as a positive control. Taken together, orthotopic GIST PDXs maintain growth capacity and properties similar to that of patients’ GIST tumors.

## Discussion

For the first time, we report an orthotopic patient-derived xenograft (PDX) model of human GIST. This model was developed in immunodeficient mice, including the NOD-scid (NS) and the NOD-scid gamma (NSG) strains. In our study, we report an 84% xenograft success rate as a proof of concept with respect to our novel approach to studying GIST. In both strains, we demonstrate that multiple intraperitoneal sites are capable of supporting GIST growth, with the liver and renal capsule allowing for high rates of engraftment. Moreover, we effectively passaged PDXs at high engraftment rates and demonstrated that high-resolution ultrasound imaging can be employed to serially follow the natural history of tumor growth. Furthermore, tumors passed from NSG mice into the renal capsules of NS mice appear to develop the most robust tumors. Finally, xenografted tumors maintain properties comparable to that of patient’s GIST tumor tissue, including cellular histology, KIT expression and FDG-avidity on PET scan.

Tumor xenografts are frequently established by subcutaneous (SQ) injection of immortalized cell lines between the dermis and underlying muscle within the flank, back or footpad of immunodeficient mice [21,33]. For over 30 years, this model has been widely used in cancer research because it is fast, cheap, reproducible, and has been considered sufficient for assessing the activity of anti-tumor agents. It also does not require expensive imaging modalities such as US, computed tomography (CT) or PET in order to visualize tumors because they can be merely measured with calipers [34]. However, these models often fail to accurately predict responses in humans since the SQ microenvironment is not relevant to the sites of primary or metastatic disease [35]. These observations have suggested that such tumor models do not represent appropriate sites for modeling human malignancies in order to evaluate responses to anti-cancer drugs [36].

Given these deficiencies in SQ models, orthotopic tumor xenografts are increasingly being utilized to develop a model with superior clinical relevance and translation applications because these models provide: 1) a biologically relevant site for tumor-host interactions; 2) the potential to develop of disease-relevant metastatic progression; 3) the ability to study site-specific dependence upon therapy; and finally, 4) organ-specific expression of genes [36]. While this approach has clear advantages as compared to SQ models, it is undoubtedly more expensive, labor intensive, technically challenging, and requires longer post-procedural healing and recovery [35,36]. Nonetheless, orthotopic tumor models have emerged as the preference for many cancer researchers.

To better approximate the genetic heterogeneity of human cancer, PDXs are now emerging as an alternative to cell lines. Like many tumors, GISTs can be SQ implanted into the flanks of mice [21-23,37,38]. However, for the aforementioned reasons, most SQ models are unable to recapitulate human tumor biology and therefore have less clinical relevance [39]. While low passage PDXs have the advantage of maintaining the tumor’s complex genetic and epigenetic abnormalities, growing them in a foreign tumor microenvironment (i.e., subcutaneously) partially negates this advantage [35]. In contrast, our xenograft model is a reproducible model of human GIST that replicates the intraperitoneal microenvironment and heterogeneity of human GISTs while allowing for the development of models (i.e., KIT exon 9 mutated GIST) that are not currently available for study in GIST cells or transgenic mouse models. Evidence also suggests that, as opposed to SQ injections, orthotopic xenografts allow for greater invasion into nearby organs, as well as, metastases to the liver [40]. In fact, we observed that GIST PDXs could grow and invade into adjacent tissues, such as the liver (Figure 5B). We have not yet observed metastases, a fact that may have been in part due to rapid local tumor progression that necessitated sacrifice of animals in compliance with IACUC regulations. Nevertheless, our model and other orthotopic xenografts provide a more pathophysiologically relevant environment for tumor growth. We believe this model can be adopted to generate new GIST models (i.e., KIT exon 9 mutants, PDGFRα mutants, and BRAF^V600E^ mutants), as well as tumors from non-gastric sites such as the small bowel, colon, rectum, esophagus, liver, and peritoneum. However, as previously noted, this model is limited by significant expense and labor utilization, as well as technical procedural challenges, the requirement for expertise in US, and the requirement for longer post-procedural healing and recovery. These factors all contributed to the modest size of our cohort.

In addition to the ability to study tumor biology, such a model can be applied for drug screening. Imatinib is considered the first line of treatment for GIST patients [41]. Unfortunately, once patients develop primary- or secondary-resistance to this drug, there are limited treatment options. One immediate potential application for our orthotopic GIST PDXs is the ability to test agents for efficacy in the setting of imatinib resistance. Hidalgo et al. reported results from their orthotopic model studies with advanced solid tumors obtained from 14 patients that were implanted into immunodeficient mice [42,43]. Once tumors were established, the mice were treated with 63 drugs in 232 treatment plans. From this murine “clinic trial,” it was determined that there exists a correlation between orthotopic PDX killing and clinical efficacy. All drugs maintained their same profile with respect to resistance and sensitivity. The data suggests that individual patient PDXs can be used to personalize a precision treatment approach to treating malignancies. Based upon our findings, a GIST phenotype can be maintained after at least 2 passages in our model. Earlier work by Revehim *et al*. demonstrated that mutations in KIT exons 11 and 17 were the same in the primary tumor and subcutaneous xenografts after multiple passages in athymic nude mice [44].

## Conclusions

In conclusion, we report the first orthotopic patient-derived xenograft model of human GIST. This novel approach provides a reproducible model of human GIST that utilizes the intraperitoneal microenvironment and maintains the genetic heterogeneity of a human gastrointestinal sarcoma. This xenograft model may enhance our ability to study GIST biology *in vivo* and serve as a preclinical platform for testing novel biomarkers and therapeutics that can inform clinical trial design.

## Abbreviations

CT: Computed tomography; DMEM: Dulbecco’s modification of eagle’s medium; DOG-1: Discovered on gastrointestinal stromal tumor 1; FDG: Fluorodeoxyglucose; FFPE: Formalin-fixed, paraffin-embedded; GIST: Gastrointestinal stromal tumor; H&E: Hematoxylin and eosin; HEPA: High-efficiency particulate air; hpf: High-power fields; IACUC: Institutional animal care and use committee; IP: Intraperitoneal; IRB: Institutional review board; NS: NOD-scid; NSG: NOD-scid gamma; PDGFRa: Platelet-derived growth factor receptor α; PDX: Patient-derived xenograft; PET: Positron emission tomography; SQ: Subcutaneous; SUV: Standardized uptake value; UCSD: University of California, San Diego; US: Ultrasonography; VEGFR: Vascular endothelial growth factor receptor

## Competing interests

Jason Sicklick received honorarium from Novartis Pharmaceuticals Corporation for advisory board consultancy and speaking, as well as reimbursement for travel, lodging, and meals. No additional authors have conflicts of interest to declare.

## Authors’ contributions

Conception and design: JKS, RS, AML. Acquisition of data: JKS, SYL, MLB, CMT, ESM, RPF, DVJ, MP. Analysis of data: JKS, SYL, CMT, CKH, MP. Interpretation of data: JKS, SYL, CMT, CKH, MP, AML. Drafting of manuscript: JKS, SYL, MLB, CMT. Critical revision of manuscript: JKS, SYL, MLB, CMT, AML. Final approval of the version to be published: JKS, SYL, MLB, CMT, ESM, RPF, DVJ, CKH, MP, RS, AML.
